# A cohort study of the recovery of health and wellbeing following colorectal cancer (CREW study): protocol paper

**DOI:** 10.1186/1472-6963-12-90

**Published:** 2012-04-04

**Authors:** Deborah Fenlon, Alison Richardson, Julia Addington-Hall, Peter Smith, Jessica Corner, Jane Winter, Claire Foster

**Affiliations:** 1 University of Southampton, Macmillan Survivorship Research Group, Southampton, UK; 2 University of Southampton, Southampton, UK; 3 University Hospital Southampton NHS Foundation Trust, Southampton, UK

**Keywords:** Colorectal cancer, Cohort study, Health and wellbeing, Survivorship

## Abstract

**Background:**

The number of people surviving colorectal cancer has doubled in recent years. While much of the literature suggests that most people return to near pre-diagnosis status following surgery for colorectal cancer, this literature has largely focused on physical side effects. Longitudinal studies in colorectal cancer have either been small scale or taken a narrow focus on recovery after surgery. There is a need for a comprehensive, long-term study exploring all aspects of health and wellbeing in colorectal cancer patients. The aim of this study is to establish the natural history of health and wellbeing in people who have been treated for colorectal cancer. People have different dispositions, supports and resources, likely resulting in individual differences in restoration of health and wellbeing. The protocol described in this paper is of a study which will identify who is most at risk of problems, assess how quickly people return to a state of subjective health and wellbeing, and will measure factors which influence the course of recovery.

**Methods/design:**

This is a prospective, longitudinal cohort study following 1000 people with colorectal cancer over a period of two years, recruiting from 30 NHS cancer treatment centres across the UK. Questionnaires will be administered prior to surgery, and 3, 9, 15 and 24 months after surgery, with the potential to return to this cohort to explore on-going issues related to recovery after cancer.

**Discussion:**

Outcomes will help inform health care providers about what helps or hinders rapid and effective recovery from cancer, and identify areas for intervention development to aid this process. Once established the cohort can be followed up for longer periods and be approached to participate in related projects as appropriate and subject to funding.

## Background

Around two million people are living with or beyond cancer in the UK and this figure is rising by more than 3% per year [1]. Whilst increasing survival rates are to be celebrated, the experiences and needs of those who have completed their primary cancer treatment have been relatively neglected [2,3]. Health professionals may be unaware of who is struggling with problems [4]. The best ways to assess problems people experience or which interventions are effective in helping relieve or prevent problems following primary treatment are largely unknown [2]. With rising numbers of survivors the need to understand problems experienced following treatment, how they can be resolved, ways in which people manage their own problems and how health professionals can support self-management are becoming increasingly important for service planners and health policy makers.

The range of problems faced by cancer survivors and how they change and resolve over time are not well understood. No published research has systematically studied the health and wellbeing of cancer survivors over the years following primary treatment [2]. US and European evidence demonstrates that cancer survivors fare less well than healthy individuals in terms of health and wellbeing [5,6], and recent UK evidence has shown that cancer survivors have similar scores to people with long term conditions on a range of measures including psychological wellbeing and physical functioning [7]. Cancer survivors have also been shown to access more health services than healthy counterparts [5,6] These studies cannot reveal the continuing care needs of individuals and whether these are met through current health care provision [2].

Failing to provide appropriate long term support across the spectrum of problems faced following primary treatment may have negative consequences for health and wellbeing of the growing number of survivors [4] and may prevent them from returning to productive lives, both socially and economically [2]. Evidence suggests that most survivors manage to live well with problems associated with cancer and its treatment; however, a substantial minority (around one third) consistently report difficulties in the long term [8]. Early intervention could help alleviate some longer term problems. For example, predicting which people are most at risk of developing problems could reduce avoidable hospital admissions [9]. In order to know how to intervene, first we must understand how health and wellbeing is restored over time (or not) and which risk and protective factors indicate who is most likely to need support and when.

Survivors of colorectal cancer form the largest group of cancer survivors affecting men and women [10]. Incidence is high (it is the third most common cancer) and survival rates have doubled in recent years with around 250,000 UK survivors [11]. Survival rates are around 52% irrespective of extent of disease at cancer diagnosis, with little decline in survival rates beyond 5 years, at which point survivors are deemed by convention to be cured [12]. As well as being a large group of survivors, colorectal cancer is treated using all the main treatment modalities of surgery, radiotherapy and chemotherapy, and so colorectal cancer patients are likely to experience many of the problems associated with cancer therapy. Available studies have consistently found that people with colorectal cancer are at risk of experiencing poor quality of life [13-18]. The cancer experience and ongoing symptoms have an impact on physical functioning and carrying out daily activities which may have long term consequences for the resumption of normal everyday life, such as return to work and finances [14,19]. Symptoms such as fatigue, psychological distress, sexual dysfunction and altered bowel habits may be long lasting [13,14,18,20]. These persistent difficulties alongside continued feelings of uncertainty and concerns for the future [21] not only have implications for an individual's health but also their sense of subjective wellbeing.

While much of the literature suggests that most colorectal cancer patients return to near pre-diagnosis status following surgery, Taylor et al [22] argue this literature has largely focused on physical side effects rather than recovery as a total human response, including emotional, spiritual and social factors, which may be equally important in the restoration of health and wellbeing [22]. Much of the work on quality of life in colorectal cancer is from cross-sectional studies [18]. Some longitudinal studies have been conducted in colorectal cancer, such as those by [23] and Taylor et al [22]. However, these have either been small scale or taken a narrow focus on recovery following cancer, such as psychological status or symptom distress [22,23]. A comprehensive, long term study exploring a comprehensive range of aspects of health and wellbeing in colorectal cancer patients and following the normal pattern of restoration of health and wellbeing has not been conducted. This paper presents the protocol for such a study, funded by Macmillan Cancer Support, to be conducted by the Macmillan Survivorship Research Group in conjunction with the University of Southampton and the National Cancer Research Network.

**Figure 1:**
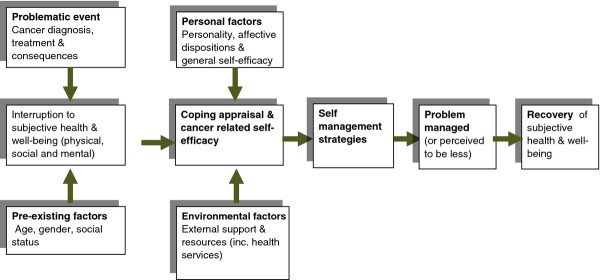
**Recovery of health and well-being in cancer survivorship **[24].

Foster and Fenlon [24] have developed a conceptual model of recovery of health and wellbeing once cancer treatment is finished, which recognises that social, physical and emotional factors all have an impact on recovery (Figure 1). The central core of the model assumes that people's subjective sense of health and wellbeing diminishes following the diagnosis and treatment of cancer and that this recovers over time. The extent to which health and wellbeing are affected and the rapidity with which they are restored will be affected by many factors. These include the severity of the illness, its treatment and subsequent impact on physical health; and also pre-existing factors, such as the age, gender and social status of the individual affected. The way in which people cope with this and work to regain their health will depend on internal factors, such as personality and self-efficacy to manage cancer related problems, and external factors, such as the support they have available to them. Our previous research suggests that confidence is key to enabling people to manage problems following primary cancer treatment and that this is important for recovery of health and wellbeing. This model will inform the data collection in the current study.

## Study aims and objectives

### Aim

The aim of the study is to establish the natural history of the recovery of health and wellbeing in people who have been treated for colorectal cancer.

### Objectives

The study objectives are to:

1. Plot the natural history of the recovery of health and wellbeing following treatment for primary colorectal cancer.

2. Investigate whether and how health needs change over this period.

3. Explore factors that influence the restoration of health and wellbeing and determine who is most at risk of poor or protracted recovery.

4. Chart utilisation of health care services in this period and explore its relationship with recovery of health and wellbeing.

5. Describe use of self-management techniques, exploring characteristics which are related to use of self-management behaviours and their relationship with recovery of health and wellbeing.

## Methods/Design

This is a prospective, longitudinal cohort study conducted in colorectal cancer survivors following primary surgery using a mailed questionnaire survey.

### Study setting

Patients will be recruited from 30 NHS treatment centres that conduct surgery for primary colorectal cancer throughout the UK. These centres will be chosen from those who express an interest through the National Cancer Research Network (NCRN). Centres will be selected for their ability to recruit high numbers of patients at a fast rate (around 2-3 per week) and able to complete their research governance procedures within 4-6 weeks. A further consideration is that centres should cover a wide range of geographical locations and ethnically diverse populations. We will also approach centres in Scotland, Wales and Northern Ireland.

### Participants

We will recruit 1000 patients with colorectal cancer through their clinical teams prior to primary surgery. Patients will be eligible for study inclusion if they: a) have a diagnosis of colorectal cancer (Dukes A-C) b) have no distant metastases c) are awaiting primary surgery (or rarely, just had primary surgery, including emergency primary surgery) d) ≥ 18 years old e) have the ability to complete questionnaires (Language line facilities will be provided for those who require it). Prior diagnosis of cancer (other than non-melanomatous skin cancer or in situ carcinoma cervix) is an exclusion criterion.

Eligible patients will be identified by a member of the patient's direct care team through the multidisciplinary team meetings (MDT) at each participating centre. All eligible patients will be allocated consecutive Research Numbers which will be documented on each site's Eligible Patient Log. The outcome for every eligible patient will be recorded against this number. Potential outcomes will be recruited, missed, declined, or recruited at a reduced consent level (see below). Patients will be invited to participate through an invitation from their medical consultant, which will be sent with the appointment letter for the patient's primary surgery pre-assessment clinic visit. Patients will be approached about study participation when attending their pre-surgical assessment appointment. At this face to face contact all eligible patients will be supplied with full study information and given a minimum of 48 hours to consider participation in the study. Study information will include details of the CREW study team at Southampton; it will emphasise the voluntary nature of participation and patients' right to withdraw consent, at any time, without the need for explanation, and without their personal care being affected. Patients who wish to join the study will be asked to read, complete and sign a consent form, which will be returned to and countersigned by the recruiting research nurse and a copy filed in patients' medical notes. Those who consent to participate will be given a baseline questionnaire which they can complete at an appropriate clinic visit or at home, and return to the CREW study team in a reply paid envelope. Patients who present as acute admissions (e.g. with bowel obstruction) will be approached by their direct care team following surgery. This group will form a separate and comparable subset of the cohort.

Consent will also be sought to inform a patient's General Practitioner (GP) of CREW study participation, including consent to contact them throughout follow-up in order to ensure inappropriate contact with a patient is avoided during the course of the study (for instance if the patient has moved, died or experienced a change in mental capacity).

Some patients may prefer not to complete questionnaires and in this case can opt to consent to allow data to be collected about them for the purposes of the research. As well as demographic details, routinely collected NHS data relevant to their disease and treatment will be supplied to the research team. Fully informed written consent will be obtained for this reduced level of study participation.

### Outcome measures

**Figure 2:**
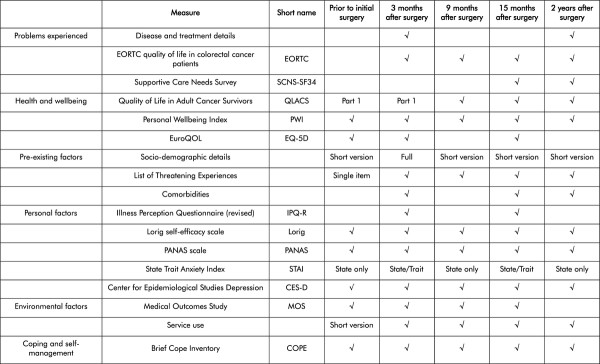
**Matrix of measures to be used in CREW cohort**.

The measures used will be informed by domains identified in our Model of Recovery (Figure 1). Not all measures will be used at each time point. QLACS will be the primary outcome measure and will be included at each time point. Other measures will be included at key times in order to answer key questions (see Figure 2).

### Problems experienced

• The cancer diagnosis and treatment are the problematic event under consideration. Data will be collected from medical records on stage, type and grade of disease as well as treatment details of surgery, radiotherapy, and chemotherapy.

• Symptom burden may also be considered part of the problematic event and this will be measured using the EORTC-QLQ-C30 (quality of life scale) and colorectal subscale EORTC-QLQ-CR29 [25]. The EORTC QLQ-C30 is a core quality of life questionnaire of 30 cancer specific items [26]. The EORTC QLQ-CR29 [25] is a colorectal cancer specific module, designed to use with the core module, of 19 items addressing gastrointestinal symptoms, pain and problems with micturition. There are separate scales for the participants with or without a stoma and separate items addressing sexual function for men and women. This scale has been widely used and demonstrates validity and reliability [27]. The Supportive Care Needs Survey (SCNS-SF34, Boyes et al 2009) will be used to capture cancer patients perceived needs using 34 questions covering five domains (psychological, health system and information, physical and daily living, patient care and support and sexuality needs). Scores are scales to range between 0 and 100, with higher scores indicating a higher level of unmet needs. Recent analysis suggests that the scale is reliable and valid when used with cancer patients [28].

### Health and wellbeing

• The Quality of Life in Adult Cancer Survivors scale (QLACS) [29] assesses quality of life in five areas suggested by long-term survivors themselves as relevant to their lives (financial problems, benefits of cancer, appearance, distress related to recurrence and distress related to the family), and seven additional areas that are relevant but not limited to cancer (negative feelings; positive feelings; cognitive problems; pain; sexual interest; sexual function; energy/fatigue and social avoidance). QLACS has been validated amongst cancer survivors [29] and has good convergent validity with other QoL measures (e.g. FACT & SF36). Part A will be used at every time point in the cohort with Part B being used at 15 months and 2 years as these questions are more relevant to long term cancer survivors.

• The Personal Wellbeing Index (PWI) [30] consists of seven items covering seven domains which represent a deconstruction of the global question "How satisfied are you with your life as a whole?"[31]. Each item is scored on 0 to 100 with a mean taken of all items, where a high score denotes high satisfaction. A score of 70 represents a threshold for failure reduced wellbeing [32]. The PWI has been found to be consistent for large populations with normative data of 74.93 for a healthy population (SD 0.75). The PWI has good reliability (Cronbach's alpha between .70 and .85, and intra-class correlation coefficient of 0.84 [33] and a high level of sensitivity between different demographic groups [31]. The EQ-5D™ is a 5 item questionnaire assessing aspects of health status followed by a 0-100 visual analogue scale asking respondents to indicate their overall health state. Health status is converted into a weighted index by applying preference weights taken from general population samples. These weights lie on an index from 0-1 in which 1 represents full health. The EQ-5D is used widely in studies of people with cancer and data is published to support its reliability and validity (Pickard et al., 2007). It provides a simple descriptive profile and a single index value for health status and is recommended for use in cost-effectiveness analyses.

### Pre-existing factors

For pre-existing factors we will collect socio-demographic details such as age; gender; marital and social status; ethnicity; living and work status. Socio-demographics which are constant, such as gender, will only be asked on one occasion, while others will be included in repeat questionnaires. Comorbid illnesses and other major life events (The List of Threatening Experiences [34] will also be collected.

### Personal factors

• The Illness Perception Questionnaire-Revised [35] measures beliefs about illness. Confidence to manage illness, or self-efficacy, will be measured using Lorig's Self Efficacy for Managing Chronic Disease [36], a 6 item scale assessing confidence to measure several items relating to chronic disease. A mean score of the items is calculated ranging from 1-10. The scale has been used in several studies measuring self-efficacy in people with cancer [37,38].

• The International Positive and Negative Affect Schedule Short Form (PANAS) [39] will be used to consider whether a positive or negative approach to life affects recover of wellbeing. The 10 question instrument consists of two 5-item mood scales, one designed to measure positive affect (PA) and the other focusing on negative affect (NA). The PANAS has been found to be a reliable and valid instrument [40] and has been used in cancer populations [41,42].

• The Center for Epidemiological Studies Depression Scale (CES-D) [43] contains 20 questions asking respondents how often they have experienced depressive symptoms. Total scores range from 0 to 60 with high scores indicating high levels of distress. A score ≥ 16 suggests a clinically significant level of psychological distress. The scale has been established as a reliable and valid instrument for measuring depressive symptoms in people with cancer [44] and has been used in several studies of this population [45,46].

• The State Trait Anxiety Inventory (STAI) [47] is a 40 item assessment which measures trait and state anxiety separately. Scores range from 20 to 80 and higher scores indicate higher levels of anxiety.

### Environmental factors

External influencing factors will be measured by the Medical Outcomes Study social support survey (MOS) [48] as well as access to services, which can be identified through postcode. The survey instrument consists of 19 questions concerning the frequency of availability of different types of support. A higher score for an individual scale or for the overall support index indicates more support available. Scale and overall scores are converted to a 0-100 value. This survey has been tested for validity and reliability among people with chronic illness [48] and has been used previously by people with cancer [49].

### Coping and self-management

Coping appraisal and coping strategies will be assessed by Carver's Brief COPE [50]. This 28 item questionnaire is organised into 14 pairs representing different coping techniques. Participants are given a score from 2 to 8 for each subscale which represents how frequently they use a particular method of coping. The questionnaire has been used in several assessments of coping styles among people with cancer [51,52], and a recent study has suggested it to be a valid and reliable instrument [53].

### Data collection

Baseline data will be collected prior to surgery. Date of surgery will be assigned as time 0. Outcome data will be collected at 5 time points: baseline (prior to initial surgery); time 1, 3 months after study entry (to monitor early adaptation and coping in those who have surgery only and detect initial treatment effects for those undergoing adjuvant chemotherapy); time 2, 9 months after surgery (those with adjuvant treatment will be completing treatment, those with surgery only may have made further progress in recovery); time 3, 15 months after surgery (those having adjuvant therapy will be in early stages of recovery); and time 4, 2 years after surgery, to assess longer term implications. Baseline data will be collected by direct approach from the recruiting clinician/research nurse. All other questionnaires will be mailed out to participants.

### Bias

Measures to reduce bias on selection include identifying eligible patients through a systematic screening process using a standardised procedure. In order to assess potential non-response bias, non-identifiable socio-demographic data (with consent) will be collected (age, gender, ethnicity, marital status, and occupation) on those who choose not to participate in the study. Where possible any reasons for non-participation will be gathered.

The questionnaire data will be self-rated, thus reducing observer bias. This will be piloted for the first three months in one centre. Strenuous efforts will be made to reduce missing data and maintain subjects in the cohort. These include GP checks to ensure patients have not moved or died, repeat questionnaires being mailed on non-response and phone calls made for missing data.

### Study size

Our sample size calculation is based on the primary outcome measure (QLACS). The mean generic summary score for QLACS is 71.2 (SD 25.6) [29]. A difference of one half a standard deviation is often quoted as a minimum clinically important difference in sample size calculations: using 80% power to detect a difference of one half of a standard deviation at the 0.05 significance level we require 46 cases in the smaller of the groups and 103 in the larger of the groups to be compared (total 149). Allowing for 30% drop-out inflates this figure to 213 [15,54]. We intend to use 25 centres, each of which will recruit an average of 40 cases. Assuming an intra-cluster correlation of 0.05 the cluster correction increases this figure to 628. We will look for a difference of one half a standard deviation for all other continuous variables and this sample size will therefore be appropriate for these variables. We have chosen to increase the sample size to 1000 cases as this will allow analysis of rarer groups.

### Analysis

For objective 1 descriptive statistics of QLACS will be used to describe changing health and wellbeing over time. We will compare this with PWI to compare this population with healthy norms and EQ5D to compare with other chronic illness populations. We will examine recurrence rates and survival curves. For objective 2 descriptive statistics of the SCNS will be used to describe changing health needs over time and comparisons made with health and wellbeing status. For objective 3 we will construct regression models to explore relationships between the recovery of health and wellbeing and factors that might influence this, such as the cancer, cancer treatments and symptom burden (EORTC QLQ-C30 + CR29), social support (MOS), personal dispositions (IPQR, PANAS and STAI), self-efficacy (Lorig), self-management (COPE), comorbidities and utilisation of health services. We will then sequence the regression models and construct graphical chain models to explore the varying contribution of all our variables. This will allow us to determine the most important factors influencing health and wellbeing outcomes and determine those most at risk of poor or protracted recovery. For objective 4 we will use descriptive statistics to demonstrate how patients access health care services over time and construct logistic regression models to explore how this relates to the recovery of health and wellbeing. For objective 5 we will describe patients' coping strategies using COPE, their confidence to self-manage problems using Lorig's self-efficacy scale and construct logistic regression models to explore how this relates to the recovery of health and wellbeing. Participants with missing data will be included in analyses for time points for which they provide data.

#### Study organisation and management

The research team and wider study advisory committee (SAC) includes lay experience of cancer and professional expertise in psychosocial oncology research, clinical oncology, nursing, data management, statistics and epidemiology. The conduct and progress of the study will be discussed and reviewed in study management meetings (SMG) fortnightly for the first six months and monthly thereafter. The SMG group will consist of the day-to-day research team and chief investigator. The SAC will meet at the beginning of the study and annually thereafter. The study is included in the National Institute for Health Research (NIHR) Clinical Research Network (CRN) Portfolio, and we will provide monthly anonymised reports on study accrual to the NIHR CRN office. We will also provide regular reports on study recruitment and progress to the colorectal and psychosocial National Cancer Research Institute clinical studies groups.

## Discussion

While much data are collected on people with colorectal cancer, the full picture of their recovery of health and wellbeing has not been captured. This study will address this issue, reporting a wide range of personal, social and physical attributes that might affect people's recovery of health following cancer. It will also give people with colorectal cancer a systematic way to record their personal perspective on the issues that affect them over a period of 2 years following surgery. Once established the cohort can be followed up for longer periods and be approached to participate in related projects as appropriate and subject to funding.

A weakness of the study is that it is not possible to gain a baseline of health prior to cancer diagnosis; the closest to baseline that can be established therefore is prior to cancer treatment. The time immediately prior to surgery has been chosen to represent baseline for a number of reasons: most people will not have had any cancer treatment at this time; most people will have had a number of planned investigations and so will be some time post diagnosis; this is a single entry point where all eligible people can be identified for inclusion in the study. Nevertheless, some people will still be too distressed, sick or frail to participate in the study. In order to account for this we have introduced a reduced level of consent so that some data is captured on this group.

This study will provide a powerful tool for understanding the process of recovery following diagnosis and treatment for cancer, to understand what factors aid or hinder people's recovery and provide evidence from which appropriate interventions can be developed to enhance the lives of people with cancer.

### Study funding and approvals

This study is funded by Macmillan Cancer Support, and is sponsored by the University of Southampton. The study protocol has received ethical and governance approvals from the National Research Ethics Service Committee South Central Oxford B (ref. 10/H1306/65), and from the University Hospital Southampton NHS Foundation Trust Research and Development Department R&D ref number: RHM CAN0737.

## Competing interests

The authors declare that they have no competing interests.

## Authors' contributions

CF and JC conceptualised the project, and obtained study funding; CF is the chief investigator, and DF, AR, JAH, PS, JC, JW are co-investigators. DF drafted and prepared the study protocol and the manuscript (which has been read and approved by all authors). DF oversees the day-to-day running of the study. All authors read and approved the final manuscript.

## Pre-publication history

The pre-publication history for this paper can be accessed here:

http://www.biomedcentral.com/1472-6963/12/90/prepub
